# Development of Freeze-Thaw Tolerant *Lactobacillus rhamnosus* GG by Adaptive Laboratory Evolution

**DOI:** 10.3389/fmicb.2018.02781

**Published:** 2018-11-20

**Authors:** Ye Won Kwon, Jae-Han Bae, Seul-Ah Kim, Nam Soo Han

**Affiliations:** Brain Korea 21 Center for Bio-Resource Development, Division of Animal, Horticultural, and Food Sciences, Chungbuk National University, Cheongju, South Korea

**Keywords:** adaptive laboratory evolution, lactic acid bacteria, *Lactobacillus rhamnosus* GG, freeze-thaw stress, genome sequencing

## Abstract

The industrial application of microorganisms as starters or probiotics requires their preservation to assure viability and metabolic activity. Freezing is routinely used for this purpose, but the cold damage caused by ice crystal formation may result in severe decrease in microbial activity. In this study, adaptive laboratory evolution (ALE) technique was applied to a lactic acid bacterium to select tolerant strains against freezing and thawing stresses. *Lactobacillus rhamnosus* GG was subjected to freeze-thaw-growth (FTG) for 150 cycles with four replicates. After 150 cycles, FTG-evolved mutants showed improved fitness (survival rates), faster growth rate, and shortened lag phase than those of the ancestor. Genome sequencing analysis of two evolved mutants showed genetic variants at distant loci in six genes and one intergenic space. Loss-of-function mutations were thought to alter the structure of the microbial cell membrane (one insertion in *cls*), peptidoglycan (two missense mutations in *dacA* and *murQ*), and capsular polysaccharides (one missense mutation in *wze*), resulting in an increase in cellular fluidity. Consequently, *L. rhamnosus* GG was successfully evolved into stress-tolerant mutants using FTG-ALE in a concerted mode at distal loci of DNA. This study reports for the first time the functioning of *dacA* and *murQ* in freeze-thaw sensitivity of cells and demonstrates that simple treatment of ALE designed appropriately can lead to an intelligent genetic changes at multiple target genes in the host microbial cell.

## Introduction

Live lactic acid bacteria (LAB) have been traditionally consumed through fermented food products and selected *Lactobacillus* strains that tolerate intestinal conditions have increasingly been used owing to their health effects. These strains, termed as “probiotics,” are described as live well-defined microorganisms with beneficial effects on wellbeing of the host (Hill et al., 2014). *Lactobacillus rhamnosus* GG (ATCC 53103) was originally isolated from the fecal samples of healthy human intestinal flora (Goldin et al., 1992). This strain possessed general characteristics of probiotics, such as tolerance to acid and bile and the ability to adhere to the intestinal epithelial layer. In addition, its diverse beneficial effects on human health, including improved intestinal health and immune systems were reported (Segers and Lebeer, 2014). Clinical treatment of *L. rhamnosus* GG showed preventive or therapeutic effects on pathogen infection such as *Escherichia coli* O157:H7 and *Salmonella* Infantis (Johnson-Henry et al., 2008; Zhang et al., 2018). Besides, the strain exhibited anti-cancer properties and mitigated the side effect of cancer therapy like diarrhea (Banna et al., 2017). These outstanding features of *L. rhamnosus* GG have encouraged its microbial studies and wide use in the industrial production of probiotic products for nearly 30 years.

The industrial exploitation and applications of LAB as starters or probiotic strains require efficient preservation technologies to maintain the viability and probiotic activities of these bacteria (Carvalho et al., 2004). Freezing preservation or freeze drying operations are widely used for the long-term storage of LAB but often negatively affect their viability (Rault et al., 2007). Exposure to low temperature and subsequent ice crystallization during freezing process are stressful to the cells. Ice crystal formation first occurs in the extracellular spaces. The withdrawal of water from the system creates a hyperosmotic extracellular environment, which in turn draws water from the cells, predominantly in the moderate freezing range down to about -20°C. As the process continues, ice crystals grow, cells shrink, and membranes and cell constituents are damaged. With further cooling, ice crystals may form within the cell, and disrupt organelles and cell membranes and cell death certainly occurs. Whether the ice is extracellular or intracellular, water is removed from the biologic system and desiccation results in cell death (Gage and Baust, 1998; Inoue et al., 2014; Beal and Fonseca, 2015). Considering the great importance of LAB in manufacturing various fermented food, the ability of the cells to be stabilized and preserved should be guaranteed. To prevent or reduce these adverse effects of freezing preservation, many substances have been used as cryoprotectants, such as skim milk, sodium ascorbate, and sugars, including trehalose, maltose, sucrose, glucose, and lactose (De Giulio et al., 2005; Jalali et al., 2012). However, apart from the use of cryoprotectants, the resistance of the cells to cold damage may effectively preserve cellular activities for a long time.

Adaptive laboratory evolution (ALE) has been used to obtain microorganisms that express the desired phenotypes (Dettman et al., 2012; Dragosits and Mattanovich, 2013). For example, piezotolerant or antibiotic resistant *E. coli* were successfully produced by ALE. In the both cases, concerted mutations occurred in the genes related to the evolved phenotype (Marietou et al., 2015; Jahn et al., 2017). ALEs of probiotics were performed for acid tolerance with *Lactobacillus casei* and *Bificobacterium longum* and they exhibited a significant increase of tolerance showing viability at lethal pH (Zhang et al., 2012; Jiang et al., 2016). While freeze-thaw (FT)-tolerant *E. coli* and industrial baker’s yeast were produced using the ALE method (Sleight and Lenski, 2007; Aguilera et al., 2010), studies have not reported the application of ALE for the production of FT-tolerant probiotics. In this study, the evolutionary adaptation method was used to obtain FT-tolerant *L. rhamnosus* GG by repeating 150 cycles of freeze-thaw-growth (FTG) for four replicates. The improvement in fitness was evaluated by comparing the extent of evolutionary adaptation of FTG-evolved mutants with that of their ancestor. In addition, genetic and phenotypic changes in FTG-evolved mutants were investigated.

## Materials and Methods

### Strain and Culture Conditions

*Lactobacillus rhamnosus* GG used in this study was obtained from the Korean Collection for Type Cultures (KCTC) with a strain number of KCTC 5033, where the strain have been sub-deposited from the American Type Culture Collection (ATCC) with strain number of ATCC 53103. *L. rhamnosus* GG (KCTC 5033) and its mutants were maintained in long-term storage at -70°C with glycerol (15% v/v) as a cryoprotectant. In FTG evolution experiment and assays for FT survival under FTG regime, glycerol was excluded to investigate the evolutionary changes in terms of survival and recovery. To ensure that cells were in physiologically comparable states at the start of FT survival and competitive fitness assays, cells were removed from the freezer, inoculated into MRS medium (Difco, Detroit, MI, United States) independently for each replicate assay, and incubated at 37°C for 24 h. The cultures were diluted 100-fold with fresh MRS broth and incubated at 37°C again for 24 h, followed by their use for all assays.

### Evolutionary Adaptation to the FTG Regime

In order to provide higher level of stress to microbial cells, we compared the survival rates of *L. rhamnosus* GG after freezing at various temperatures for 6 h. As results, at deep-freezing temp (-80°C), the survival rate was relatively high (>70%) revealing lower level of freezing stress to microbial cells, possibly due to the formation of small size ice crystal (Gilkey and Staehelin, 1986). Thereby, we increased the temperature gradually and found that -30°C was proper to give high level of freezing stress (60% survival rate) as well as fast freezing (6 h). Meanwhile, -18°C resulted in the same level of freezing stress (60% survival rate) but slower freezing rate (>6 h). Evolutionary experiment was processed in 1 mL of MRS medium using Eppendorf tubes with four replicates from the original ancestor *L. rhamnosus* GG KCTC5033. It was performed with a regime of 1 day of freezing at -30°C for 6 h (without added cryoprotectant) and thawing at 13°C for 2 h, followed by growth in a fresh medium after 1% inoculation at 37°C for 16 h. After 1-day cycle of freezing-thawing-growing (FTG), the 2nd cycle was repeatedly carried out. These four populations evolved for 150 times of 1-day FTG cycles, where 1 day corresponds to one cycle. During the FTG cycles, examination of contamination was performed every month using 16S rRNA gene sequence analysis. After 70, 100, 130, and 150 FTG cycles, colonies were isolated on MRS agar medium, and their FT survival rates and growth dynamics were analyzed to select single colony representing evolved replicates.

### Calculation of FT Survival

For the analysis of survival rates of evolved replicates and ancestor, viable cell density of each population was measured after FT treatment. After thawing the frozen cells, they were serially diluted and appropriate volume of the cells were spreaded on MRS agar medium. After incubation at 37°C for 48 h, the numbers of colonies were counted. FT survival rate (*S*) was calculated as follows:

$$S=\;\frac{N_{1}}{N_{0}}\times 100\;$$

where *N*_1_ is the viable cell density measured after thawing the frozen cells and *N*_0_ is its initial cell density before freezing. Relative FT survival was expressed as a ratio compared to the survival rate of ancestor.

### Growth Dynamics

Separate growth curves were obtained based on optical density (OD) at 600 nm for the evolved cells and their ancestor *L. rhamnosus* GG after treatment of 150 FTG cylcles for 24 h using a microplate spectrophotometer (PowerWave HT, BioTek, United States) to compare their growth dynamics at 37°C. OD values were measured every hour during lag phase, every 2 h from exponential phase to early strationary phase, and at 24 h. Other growth curves of *L. rhamnosus* GG were obtained at a 10-min interval from the start of the culture to the middle of exponential phase to estimate durations of their lag phases before growth commencement as well as their doubling times during their growth phases. The duration of lag phase was calculated using the general method given by Sleight and Lenski (2007). OD values from growth curve experiments were standardized by dividing by the initial OD measured immediately after the FT cycle before dilution and log_2_ transformed in order to express changes as doublings. The transformed data were plotted and inspected to identify exponential growth, and linear regression was performed on the identified exponential-phase data. The doubling time was calculated as the inverse of the slope of regression. The “apparent” lag phase was estimated by extrapolating the exponential growth back in time until the regression intersected with the initial OD measurement.

### Whole Genome Resequencing

The genomes of the two evolved (LR1 and LR2) and ancestor *L. rhamnosus* GG strains were sequenced after 150 FTG cylcles. The evolved mutants were selected by single colony isolation on MRS agar medium, followed by confirmation of improved fitness. For each strain, genomic DNA was extracted using the genomic DNA prep kit (SolGent, Korea) for library construction, and DNA purity was determined by recording the ratio of absorbance at 260/280 and 260/230 nm using the Epoch plate reader (BioTek Instrument Inc., Winooski, VT, United States). The genomic DNA libraries were conctructed using TruSeq DNA PCR-free kit (Illumina, CA, United States). The DNAs were randomly fragmented and repaired to blunt end with end repair enzyme included in the kit. After A-tailing, 5′ and 3′ adapters were ligated. The genomes were sequenced with a sequencer, HiSeq4000 (Macrogen Inc., Korea). After quality control and quality filtering process, sequence reads were mapped first using *L. rhamnosus* ATCC 53103 as the reference genome (GenBank accession number NC_013198.1) (Morita et al., 2009) and next *L. rhamnosus* KCTC 5033 (GenBank accession number: CP031290) for correction of natural mutations with BWA (ver.0.7.12) program to produce aligned reads. After mapping, duplicated reads were removed with Picard (ver. 1.119) and variant calling were performed using SAMTool (ver. 1.2). The results were analyzed by comparing the sequences in the NCBI^1^ and BioCyc^2^ databases.

### Statistical Analysis

All experiments were conducted in triplicate, and the results were presented as the mean ± standard deviation. Statistical analysis for significant differences was performed using one-way ANOVA in Minitab.

## Results

### Evolutionary Adaptation to the FTG Regime

**FIGURE 1:**
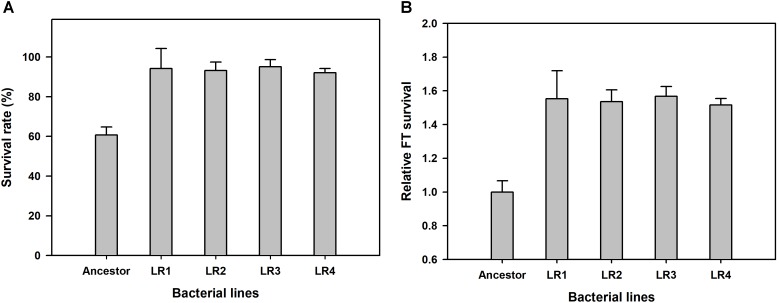
Overall fitness gains after 150 cycles of freeze-thaw-growth (FTG) regime in terms of changes in survival rate **(A)** and freeze-thaw (FT) survival **(B)** of FTG-evolved mutants and their ancestral *Lactobacillus rhamnosus* GG strain. The four evolved mutants designated as LR1 to LR4 evolved for 150 FTG cycles. Error bars are standard deviation from three replicates. Values with different letters are significantly different (*P* < 0.05).

We performed ALE experiment under the FTG regime for 150 cycles, and compared the relative fitness of four FTG-evolved mutants and their ancestral strain. Survival rates were measured after FT treatment for 150 cycles, and they were calculated by measuring the viable cell density before and after FT treatment to show improved fitness under the FTG. As shown in Figure 1A, the mean survival rates of four evolved mutants were increased up to 93.65% after FT treatment from 60.69% (before FT treatment) showing great enhancement resulted from FTG evolution. As same, the overall fitness gains of four evolved mutants were 54% in terms of relative freeze-thaw survival compared to the ancestor strain (Figure 1B). Their increased fitness gains were all significant.

**FIGURE 2:**
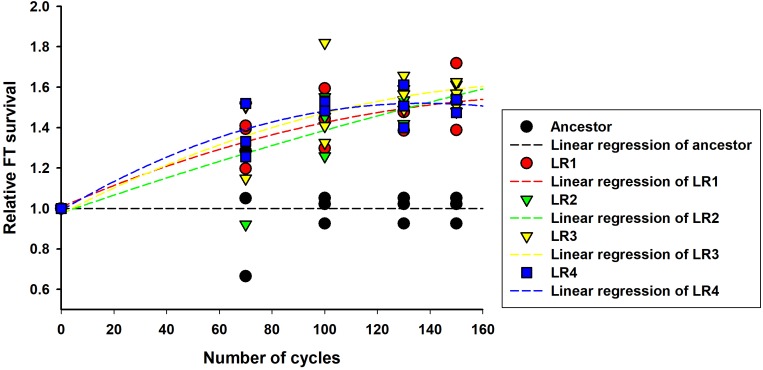
Rates of evolution and FT survival improvement in FTG-evolved *L. rhamnosus* GG.

**FIGURE 3:**
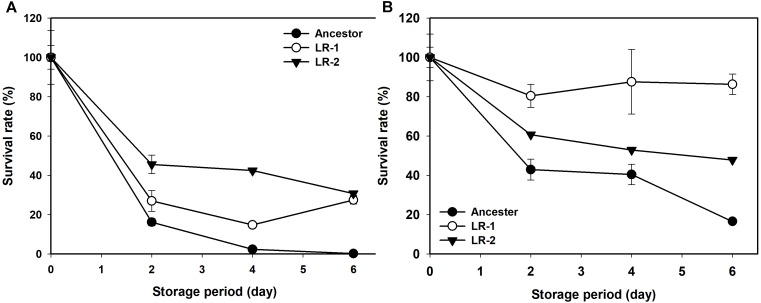
Changes in survival rate of FTG-evolved mutants and their ancestral *L. rhamnosus* GG strain during frozen preservation at –30°C in MRS with no cryprotectant **(A)** and in MRS with 0.3 M trehalose as cryprotectant **(B)**. The two evolved mutants designated as LR1 and LR2 are evolved for 150 FTG cycles. Error bars are standard deviation from three replicates.

Next, the rates of evolution and FT survival improvement of evolved mutants were measured after 70, 100, 130, and 150 cycles of FTG treatment (Figure 2). The four evolved mutants showed a rapid increase in relative FT survival during the initial stage of FTG treatment, but the rates of increase were gradually slowed down after 100 cycles; particularly LR4 mutant exhibited a decrease in the relative FT survival rate, thereafter. As shown in Figure 3, during the frozen storage at -30°C for 6 days, the survival rates of 150 cycle-evolved mutants (LR 1 and LR 2) and ancestor were compared. When cells were frozen in MRS medium without trehalose as a cryoprotectant, the freezing stress at -30°C for 6 days was lethal to the ancestral strain showing 0.2% survival rate, while the evolved mutants, LR 1 and LR 2, were tolerant to the stress showing 27.4 and 30.7% of survival rates, respectively. When those cells were frozen with 0.3 M trehalose, the ancestor obtained low level of tolerance (16.5%) after 6 days, while LR-1 and LR-2 showed significant increase of tolerance, 86.3 and 47.8%, respectively. This result reveals that the evolved mutants after 150 cyles of FTG regime, obtained an improved fitness (survival rates) to the condition of frozen storage.

### Changes in Growth Dynamics

**FIGURE 4:**
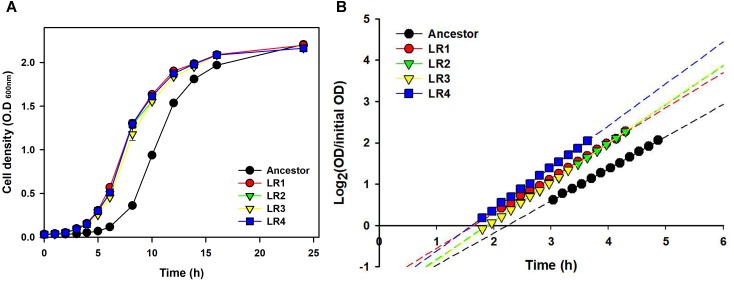
Growth dynamics **(A)** and lag phases **(B)** following FT treatment for four FTG-evolved mutants and their ancestor *L. rhamnosus* GG.

To compare growth dynamics of the evolved mutants and their ancestral strain after 150 cycles of FTG, two different types of growth curves were obtained (Figure 4). Figure 4A shows the growth dynamics, based on the OD, for four evolved mutants and their ancestor after FT treatment. Differences in the growth dynamics between these strains were apparent following the FT cycle. All evolved groups showed faster growth than their ancestor. Figure 4B shows the doubling time during the exponential growth phase and apparent lag duration calculated as described in the Section “Materials and Methods.” The evaluation of growth curves after FT treatment showed that the evolved mutants showed much faster exponential growth rates, with a 13-min shorter mean doubling time than their ancestor. The apparent lag phase of the ancestor was 134 min, while the mean apparent lag phase for the evolved mutants was only 105 min, indicating a difference of 29 min (Figure 4B).

### Genome Sequence of *L. rhamnosus* GG

In this study, we used *L. rhamnosus* GG obtained from the KCTC with a strain number of KCTC 5033, where the strain have been sub-deposited from the ATCC with strain number of ATCC 53103. We analyzed the whole genome sequence of *L. rhamnosus* GG KCTC 5033 and mapped using *L. rhamnosus* ATCC 53103 as the reference genome (GenBank accession number NC_013198.1). We found 41 SNPs in KCTC 5033 strain (Table 1). The sequences of *L. rhamnosus* GG KCTC 5033 were deposited in GenBank (accession numbers: CP031290).

**Table 1:** Identification of SNPs in genome of *Lactobacillus rhamnosus* GG KCTC 5033 compared with that of *L. rhamnosus* GG ATCC 53103 as the reference genome.

No	Start	End	Strand	Mutation Position	Reference sequence	Altered sequence	Amino acid change	Enzyme
1	263021	264508	++	264278	G	A	Ala→Thr	MFS transporter
2	614219	616219	+	615483	T	C	Leu→Pro	PTS beta-glucoside transporter subunit IIABC
3	655200	657050	-	656560	ACGCCGCC	ACGCCGCCGCC	QGGV→ QGGGV	alpha-glycerophosphate oxidase
4	Intergenic space		/	751602	G	A	/	/
5	Intergenic space		/	751632	T	G	/	/
6	Intergenic space		/	751633	T	C	/	/
7	Intergenic space		/	751663	T	C	/	/
8	876317	876952	-	876397	TGCCGCCG	TGCCGCCGCCG	LPGGI→ LPAGGI	Copper homeostasis protein CutC
9	Intergenic space		/	1154682	T	G	/	/
10	1370968	1372521	+	1372183	T	A	Leu→Met	2′, 3′-cyclic nucleotide 2′-phosphodiesterase
11	Intergenic space		/	1873061	CGG	CG	/	/
12	Intergenic space		/	1873072	G	T	/	/
13	Intergenic space		/	1873075	C	G	/	/
14	Intergenic space		/	1873076	T	C	/	/
15	Intergenic space		/	1873089	G	A	/	/
16	Intergenic space		/	1873091	T	C	/	/
17	Intergenic space		/	1873092	G	A	/	/
18	Intergenic space		/	1873879	T	C	/	/
19	Intergenic space		/	1883242	C	A	/	/
20	1993822	1997424	-	1994717	T	A	Asp→Val	peptidoglycan-binding protein LysM
21	2158636	2160858	+	2158707	C	T	X	BREX-1 system adenine-specific DNA-methyltransferase
22	2158636	2160858	+	2158710	G	A	X	
23	2158636	2160858	+	2158719	C	T	X	
24	2161973	2165527	-	2164868	T	C	X	BREX-1 system adenine-specific DNA-methyltransferase
25	2161973	2165527	-	2164871	A	G	X	
26	2351546	2355847	-	2352806	G	A	X	Cell surface protein
27	2351546	2355847	-	2352965	A	T	X	
28	2351546	2355847	-	2353053	A	G	Val→Ala	
29	2351546	2355847	-	2353148	T	C	X	
30	2351546	2355847	-	2353155	T	C	Gln→Arg	
31	2351546	2355847	-	2353178	G	A	X	
32	2351546	2355847	-	2353217	A	G	X	
33	2351546	2355847	-	2353220	A	T	X	
34	2351546	2355847	-	2353382	G	A	X	
35	2351546	2355847	-	2353391	C	T	X	
36	2351546	2355847	-	2353466	G	A	X	
37	2351546	2355847	-	2353548	G	A	Ser→Phe	
38	2351546	2355847	-	2353713	A	G	Val→Ala	
39	2452189	2453244	+	2452317	A	G	X	Hypothetical protein
40	2574214	2574357	+	2574344	A	G	Glu→Gly	Malolactic regulator
41	2574214	2574357	+	2574345	A	G	X	

### Analyses of Genetic Changes

To compare the genetic changes in the evolved mutants, genome sequences of the two FTG-evolved mutants (LR1 and LR2) were analyzed and compared with that of *L. rhamnosus* KCTC 5033. As presented in Table 2, the mutations in the FTG-evolved mutants were identified both in six gene regions and an intergenic space. Single-nucleotide polymoprphism (SNP) mutations in the cardiolipin synthase gene were detected in both LR1 and LR2 mutants at different gene loci. The gene encoding exopolysaccharide biosynthesis protein showed sequence variations at two different positions within the same gene region. BREX-1 system adenine-specific DNA-methyltransferase gene was mutated at two gene regions that encoded the same protein. The gene encoding 2-nitropropane dioxygenase also showed the same mutation at the same position in both mutant strains. The mutations in the genes encoding D-alanyl-D-alanine carboxypeptidase and *N*-acetylmuramic acid-6-phosphate etherase were found only in the LR2 mutant. The mutation in the intergenic space was located between LGG_RS07350 gene, encoding a terminase, and LGG_RS07355 gene, encoding a recombinase.

**Table 2:** Genetic mutations identified in FTG-evolved *L. rhamnosus* GG clones.

Position (bp)	Gene or region	Gene description	Mutation	Identification of mutation	
				LR 1	LR 2
256855	LGG_RS01240 (*dacA*)	D-Alanyl-D-alanine carboxypeptidase	Insertion (position 903, +A)	×	○
1272968	LGG_RS06120 (*cls*)	Cardiolipin synthase	Missense	○	×
1473969	LGG_RS07040 (*cls*)			×	○
2106059	LGG_RS09880 (*wze)*	Exopolysaccharide biosynthesis protein	Missense	×	○
2106381				○	×
2160010	LGG_RS10085 (*pglX*)	BREX-1 system adenine-specific DNA-methyltransferase	Missense	○	○
2163622	LGG_RS10095 (*pglX*)			○	○
2192576	LGG_RS10195 (*fabK*)	2-nitropropane dioxygenase	Missense	○	○
2844194	LGG_RS13275 (*murQ*)	*N*-acetylmuramic acid-6-phosphate etherase	Missense	×	○
1551606	LGG_RS07350← / ←LGG_RS07355	Intergenic space	Intergenic mutation (-117/+365, G to T)	○	○

## Discussion

*Lactobacillus rhamnosus* GG (ATCC 53103) is one of the most studied probiotic strains that has shown to give beneficial effects to host. Probiotic foods should be safe and contain appropriate probiotic organisms in sufficient number at the time of consumption. Therefore, the probiotic strain selected must be suitable for large-scale industrial production and should be able to survive and retain its functionality during production and storage as frozen or dried culture (Tripathi and Giri, 2014). Freezing has been generally used to preserve starter cultures for years because it effectively inhibits the activities of spoilage microorganisms and maintains the starter cultures for a long time (Archer, 2004; Barria et al., 2013). However, ice crystal formation may occur in the extracellular spaces during freezing and cause cell destruction. Thus, development of FT-tolerant probiotic microorganisms is desirable for their safe and effective applications in food industries.

We produced four evolved mutants that showed FT tolerance. All mutant strains exhibited enhanced fitness including improved survival rates, faster growth rate, and shortened lag phase and doubling time than those of the ancestor (Figures 1–4). This result is comparable with those of previous reports, wherein the evolutionarily adapted strains of *E. coli* and industrial baker’s yeast subjected to FT stress showed improved fitness after ALE using the FTG regime or growth at low temperature (Sleight and Lenski, 2007; Aguilera et al., 2010). The FT survival representing evolution rates increased rapidly at the initial FTG treatment, but slowed down after 100 cycles. The same trend was observed in other microorganisms such as in *E. coli* and yeast; previous studies have reported the rapid increase in fitness observed during the early phase that eventually slows down during the extended evolution in ALE experiments (Sleight and Lenski, 2007; Aguilera et al., 2010). This phenomenon also accompanies accumulation of random mutagenesis along with the evolutionary cycles that would result in network complexity to lose intrinsic microbial attributes (Hua et al., 2007; Barrick et al., 2009).

Genetic changes of *L. rhamnosus* GG during the ALE procedure were determined through the comparison of the genome sequences of FTG-evolved strains and their ancestor. In this procedure, we identified 41 SNPs in the genome of KCTC 5033 ancestor strain compared with the genome of ATCC 53103 strain (Table 1). Those single mutations are supposed to have been occurred randomly during the preservation period in the KCTC after sub-deposition from the ATCC. This result reveals that most genome sequences of stock cultures harbored in culture collection centers or laboratories might be changed by random mutagenesis along with preservation period. Mutations in the evolve mutants were identified in six genes and in one intergenic space (Table 2). Most mutated genes were involved in the biosynthesis of the cell wall or membrane (Hirschberg and Kennedy, 1972; Shibuya and Hiraoka, 1992; Jaeger and Mayer, 2008; Mohammadi et al., 2011; Liechti et al., 2014; Kang et al., 2015). In addition, the mutation in the intergenic space may affect the regulatory sequence of the gene region. The mutations in the evolved lines may disrupt the function of the encoded enzymes and consequently cause structural changes in cell membranes and walls, resulting in an increase in the cellular fluidity that is known to benefit cells by providing resistance against FT stress. In biology, membrane fluidity refers to the viscosity of the lipid bilayer of a cell membrane or a synthetic lipid membrane (Gennis, 1989).

The genes encoding D-alanyl-D-alanine carboxypeptidase (DacA) and *N*-acetylmuramic acid-6-phosphate etherase (MurQ) are involved in peptidoglycan (PG) synthesis. PG is the major component of the gram-positive bacterial cell wall and ensures cell wall rigidity and stability. PG is a macromolecule comprising glycan chains made up of alternating β-1,4-linked *N*-acetylglucosamine (GlcNAc) and *N*-acetylmuramic acid (MurNAc) cross-linked with oligopeptides at the lactic acid residue of MurNAc. During PG biosynthesis, a dipeptide ligated with two D-alanine by D-alanine-D-alanine ligase is first added to the tripeptide in PG stem by MurF resulting in a pentapeptide. Thereafter, the terminal D-alanine is cleaved from the stem peptide catalyzed by DacA (EC 3.4.16.4) and the resulting tetrapeptide is cross-linked with adjacent peptide stems by transpeptidases (penicillin binding proteins) (Liechti et al., 2014). Furthermore, MurQ (EC 4.2.1.126, MurNAc-6-P etherase) is known to catalyze the cleavage of MurNAc 6-phosphate, with GlcNAc 6-phosphate and d-lactate involving in the recycle of PG (Jaeger et al., 2005; Jaeger and Mayer, 2008). While no experimental evidence of the reverse reaction by this enzyme to synthesize MurNAc 6-phosphate connecting PG between glycan and peptide, MurQ is supposed to be one of the core enzyme for PG metabolism. Mutations at *dacA* and *murQ* genes in LR1 and LR2 may cause changes (possibly decrease) in the enzymatic activity and affect the crosslinking between peptide chains and alteration in the synthesis (or recycle) of PG, consequently leading to a decrease in cell wall rigidity.

Cardiolipin (CL) synthase (encoded by *cls* gene) is involved in the synthesis of the cellular compartment of lipid bilayer. It converts two phosphatidylglycerol molecules into CL and glycerol in the membrane during stationary phase (Hirschberg and Kennedy, 1972; Shibuya and Hiraoka, 1992). CL is a dominant phospholipid in stationary-phase cells and functional defects in *cls* gene can inhibit CL synthesis and PG accumulation during stationary phase (Bauman et al., 1965; Houtsmuller and Van Deenan, 1965; Pluschke et al., 1978; Pluschke and Overath, 1981). Previous studies reported that *cls E. coli* mutant altered membrane phase transitions during or after temperature downshift, resulting in a significant increase in the membrane fluidity, while other *cls*^-^ mutants represented improved FT survival and growth rate after FT treatment (Pluschke and Overath, 1981; Sleight et al., 2008). As same, in our study, it was found that the *cls* gene may be involved in the membrane structure of *L. rhamnosus* GG.

The protein Wze or CpsD is involved in the biosynthesis of exopolysaccharide (EPS) or capsular polysaccharides (CPS) in bacteria, respectively. A phosphorylation complex containing Wze (CpsD homolog), an autophosphorylating tyrosine kinase, and Wzb (CpsB), a phosphotyrosine protein phosphatase, is thought to be involved in the regulation of EPS biosynthesis (Lebeer et al., 2009). This mechanism was proved by Toniolo et al. (2015) by performing a mutational study for each gene associated with the biosynthesis of CPS in *Streptococcus agalactiae*. CpsA was found responsible for the transfer of CPS from the membrane lipid to cell wall peptidoglycan, and this process was controlled by CpsC that inhibits the action of CpsA. CpsC is inhibited by the activation of CpsD, resulting in CpsA activation and CPS transfer. The loss-of-function mutation in *wze* (CpsD) fails to inhibit CpsC and this change results in the continuous elongation of CPS on the membrane.

**FIGURE 5:**
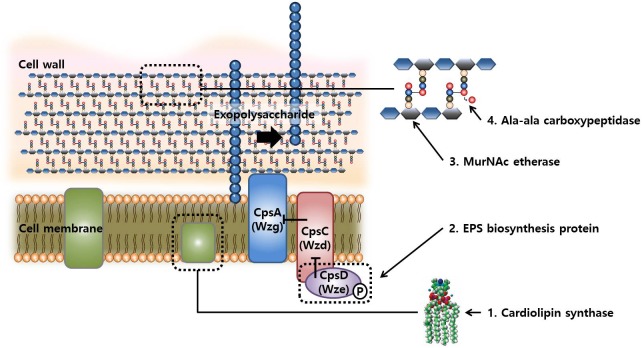
Schematic representation of the compartment synthesis in cellular structures and possible synthetic reactions involved in changes in FT-tolerance by mutations.

Taken together, the mutations in the evolved strains after FT treatment mainly occurred in the genes related to the biosynthesis of cell membrane, cell wall, and capsular polysaccharides. The hypothetical mechanisms underlying these mutations are illustrated in Figure 5. The cell envelope is rigid and highly structured to maintain the cell structures and protects cells from environmental stress. However, functional defects in these genes may produce incomplete cell wall or membrane, resulting in a flexible cell envelope that may be beneficial in FT stress because these cells are more tolerant to the pressure of ice crystal formation in intra- and extracellular space as compared with the wild-type cells.

Additional mutations were identified in two genes and in one intergenic space. One of the mutated genes was that encoding 2-nitropropane dioxygenase (FabK), an enzyme catalyzes the oxidation of nitroalkanes into their corresponding carbonyl compounds and nitrite, and the other gene encoded BREX-1 system adenine-specific DNA-methyltransferase (*pglX*), which can be involved in the phage resistance system. The genes adjacent to the mutation point of intergenic space were those encoding the phage terminase small subunit P27 family and recombinase family protein. Phage terminase small subunit P27 family is an enzyme that creates cohesive ends during DNA packaging, while the recombinase family protein catalyzes DNA recombination for the manipulation of genome structure and control of gene expression. However, a possible association between these genes and FT stress is not clear yet.

## Conclusion

In conclusion, we were able to select the FT-tolerant *L. rhamnosus* GG mutants (4 lines) by ALE repeating a day-FTG regime. After FT treatment for 150 cycles, the survival rates of mutants were significantly improved: reaching up to >90% from 60%. Genome sequencing of two mutants showed six SNP mutations occurred in their chromosome and they were related to the biosynthetic mechanisms of cellular membrane-wall-EPS structure. Incomplete or altered formation of cellular envelope might result in enhancement in its flexibility, which plays important roles in the microbial FT tolerance. In this study, we report, for the first time, the effect of mutations in *dacA* and *murQ* genes related with freeze-thaw tolerance of cells. This study demonstrates a feasibility of ALE to select microorganisms possessing preferred phenotypes caused by genetic changes in multiple genes located distant loci of chromosomal DNA.

## Author Contributions

NH conceived and supervised this study. YK performed the experiments and generated the draft manuscript. All authors contributed to data interpretation. J-HB and S-AK contributed organizing data, statistical analysis, gene annotation, and writing the manuscript. All authors reviewed and approved the final manuscript.

## Conflict of Interest Statement

The authors declare that the research was conducted in the absence of any commercial or financial relationships that could be construed as a potential conflict of interest.

## Footnotes

1 www.ncbi.nlm.nih.gov

2 https://biocyc.org/
